# High expression of protein tyrosine phosphatase receptor S (PTPRS) is an independent prognostic marker for cholangiocarcinoma

**DOI:** 10.3389/fpubh.2022.835914

**Published:** 2022-08-01

**Authors:** Muntinee Lertpanprom, Atit Silsirivanit, Patcharaporn Tippayawat, Tanakorn Proungvitaya, Sittiruk Roytrakul, Siriporn Proungvitaya

**Affiliations:** ^1^Centre of Research and Development of Medical Diagnostic Laboratories, Faculty of Associated Medical Sciences, Khon Kaen University, Khon Kaen, Thailand; ^2^Cholangiocarcinoma Research Institute, Khon Kaen University, Khon Kaen, Thailand; ^3^Department of Biochemistry, Faculty of Medicine, Khon Kaen University, Khon Kaen, Thailand; ^4^Functional Ingredients and Food Innovation Research Group, National Center for Genetic Engineering and Biotechnology, National Science and Technology Development Agency, Pathumthani, Thailand

**Keywords:** cholangiocarcinoma, proteomics, bioinformatics, PTPRS, serum

## Abstract

Cholangiocarcinoma (CCA) is an aggressive tumor of the bile duct with a high rate of mortality. Lymph node metastasis is an important factor facilitating the progression of CCA. A reliable biomarker for diagnosis, progression status, or prognosis of CCA is still lacking. To identify a novel and reliable biomarker for diagnosis/prognosis of CCA, liquid chromatography-mass spectrometry and tandem mass spectrometry (LC-MS/MS) in combination with bioinformatics analysis were applied for the representative serum samples of patients with CCA. The proteome results showed that protein tyrosine phosphatase receptor S (PTPRS) had the highest potential candidate. Then, a dot blot assay was used to measure the level of serum PTPRS in patients with CCA (*n* = 80), benign biliary disease patients (BBD; *n* = 39), and healthy controls (HC; *n* = 55). PTPRS level of CCA sera (14.38 ± 9.42 ng/ml) was significantly higher than that of BBD (10.7 ± 5.05 ng/ml) or HC (6 ± 3.73 ng/ml) (*P* < 0.0001). PTPRS was associated with serum albumin (*P* = 0.028), lymph node metastasis (*P* = 0.038), and the survival time of patients (*P* = 0.011). Using a log-rank test, higher serum PTPRS level was significantly (*P* = 0.031) correlated with a longer overall survival time of patients with CCA, and PTPRS was an independent prognostic marker for CCA superior to carbohydrate antigen 19-9 (CA19-9), carcinoembryonic antigen (CEA) or alkaline phosphatase (ALP). High expression of PTPRS could be a good independent prognostic marker for CCA.

## Introduction

Cholangiocarcinoma (CCA) is a malignant tumor arising from the bile duct epithelium and it is one of the serious health problems in endemic areas due to its poor survival rate. The global incidence of CCA is 0.3–6.0 per 100,000 people, and the highest CCA incidence rate reported in the world is 118.5 per 100,000 people in Khon Kaen, our study area, in Northeast Thailand (1–3). The incidence rate has been increasing in the past few decades worldwide, representing a global health problem (1). In Southeast Asia, *Opisthorchis viverrini* infection through consumption of undercooked fish is the major risk factor for CCA. Related to this, CCA seen in the greater Mekong subregion including our study area is rather a homogenous phenotype of intrahepatic adenomatous or papillomatous type. Because of the intrahepatic nature, most patients with CCA are asymptomatic at the early-stage of the disease (4, 5). Serum tumor markers such as carcinoembryonic antigen (CEA), carbohydrate antigen 19-9 (CA19-9), and liver function tests including serum bilirubin, alkaline phosphatase (ALP), and gamma-glutamyl transferase (GGT) are the most widely used markers for primary screening of CCA in routine clinical laboratory examinations (6, 7).

Lymph node (LN) metastasis is commonly seen in CCA. In fact, 45% of patients with CCA were already found to have LN metastasis at the time of surgical treatment. More than half of patients with CCA without LN metastasis are asymptomatic. In intrahepatic CCA, the 5-year survival of patients with or without LN metastasis was 0–9% and 36–43%, respectively. Surgery is a primary choice of treatment for CCA (1, 8, 9). However, most patients are diagnosed at the late stage with metastasis and require chemotherapy. The current first-line therapeutic treatment for advanced CCA is a cisplatin-gemcitabine combination based on the ABC-02 trial data. In spite of the efforts, the prognosis of patients is poor with an overall survival (OS) of <12 months. Recently, immunotherapy for CCA, as a monotherapy or in combination with anticancer agents, has been developed and its efficacy is evaluated in clinical trials (10–12). Therefore, regardless of the therapeutic approach, a reliable method for early diagnosis and prediction of prognosis is necessary to improve the quality of life of CCA.

In recent years, proteomic analysis using a high-throughput technique like mass spectrometry (MS) has been used in system biology and applied medical research. Proteomic analysis has been applied for biomarker discovery and many protein biomarker candidates have been successfully discovered based on MS analysis (13, 14). In biomarker search for CCA using a proteomic approach, we previously reported that apurinic/apyrimidinic endodeoxyribonuclease 1 (APEX1) as a diagnostic and prognostic biomarker for CCA (14). Likewise, our group also identified pyruvate dehydrogenase kinase (PDK) as a potential prognostic marker for CCA (15). The coupled tool of liquid chromatography (LC) and MS provides high sensitivity and specificity for protein identification and quantitation (16, 17). The aims of this study are to identify a novel diagnostic/prognostic biomarker in the sera of patients with CCA using LC-MS/MS and evaluate its clinical applicability for the prediction of LN metastasis.

## Materials and methods

### Serum samples and ethics statement

This study was approved by the Human Ethics Committee of Khon Kaen University (approval no. HE631337) and written informed consent was obtained from each of the participants.

Serum samples of 80 cholangiocarcinoma (CCA) and 39 benign biliary diseases (BBD) patients consisting of cholangitis, cholecystitis, chronic inflammation, and cholelithiasis were provided from the Cholangiocarcinoma Research Institute (CARI), Faculty of Medicine, Khon Kaen University, Khon Kaen, Thailand. The nodal status of CCA in this study was based on pathological findings collected from the patients' records provided by CARI. Among 80 patients with CCA, 38 were with LN metastasis and 38 were without LN metastasis, while four were unknown about metastasis. The inclusion criteria for patients with CCA selection were the patients being diagnosed as having intrahepatic CCA by pre-operative pathological examinations, while the exclusion criteria for patients with CCA selection were the patients being diagnosed as having extrahepatic CCA or hepatocellular carcinoma. The clinical laboratory data including biochemical tests and serum tumor markers (CEA and CA19-9) were obtained from the patient's record database of CARI. In addition, 55 serum samples of healthy controls (HC) were obtained from the leftover sera of healthy persons who received the annual health check-up in the Faculty of Associated Medical Sciences (AMS-KKU Excellence Laboratory), Khon Kaen University, whose general appearance and liver function are within normal range. All serum samples were stored at −20°C until use.

### Sample preparation and trypsin digestion

For the selection of candidate proteins, as the first step, three samples each were randomly selected from HC, BBD, and CCA with and without LN metastasis groups. A total of 12 (three samples × four groups) serum samples were subjected to LC-MS/MS analysis. For tryptic digestion, 4 μg protein from each serum sample was processed using an in-house method developed by the Functional Proteomics Technology Laboratory, National Center for Genetic Engineering and Biotechnology (BIOTEC), Thailand. In brief, serum samples were directly congelated in the microtube by mixing with a solution composed of 40% acrylamide/bisacrylamide, 1.5 M Tris-HCl (pH 8.8), 10% SDS, 10% ammonium persulfate, and tetramethylethylenediamine (TEMED) followed by centrifugation at 10,000 × g for 5 min. After incubation for 5 min at room temperature, the gel was formed and the gel pieces were dehydrated in 100% acetonitrile, reduced with 5 mM dithiothreitol (DTT) in 10 mM ammonium bicarbonate (AMBIC), and alkylated with 15 mM iodoacetamide (IAA) in 10 mM AMBIC. Then, tryptic digestion was performed overnight at 37°C. The digested peptides were extracted, dried, and stored at −80°C.

### Liquid chromatography-tandem mass spectrometry (LC-MS/MS)

The tryptic peptide samples were prepared for injection into an Ultimate3000 Nano/Capillary LC System (Thermo Scientific, UK) coupled to a Hybrid quadrupole Q-Tof impact II™ (Bruker Daltonics) equipped with a Nano-captive spray ion source. Briefly, peptides were enriched on a μ-Precolumn 300 μm i.d. × 5 mm C18 Pepmap 100, 5 μm, 100 A (Thermo Scientific, UK), separated on a 75 μm I.D. × 15 cm, and packed with Acclaim PepMap RSLC C18, 2 μm, 100 Å, nanoViper (Thermo Scientific, UK). Solvents A and B containing 0.1% formic acid in water and 0.1% formic acid in 80% acetonitrile, respectively were supplied on the analytical column. A gradient of 5–55% solvent B was used to elute the tryptic peptides at a constant flow rate of 0.3 μl/min for 30 min. Electrospray ionization was carried out at 1.6 kV using the CaptiveSpray. Mass spectra (MS) and MS/MS spectra were obtained in the positive ion mode over the range (m/z) 150–2,200 (Compass 1.9 software, Bruker Daltonics). The LC-MS analysis of each sample was done in triplicate.

### Protein quantitation and identification

MaxQuant 1.6.6.0 was used to quantify the proteins in individual samples using the Andromeda search engine to correlate MS/MS spectra to the Uniprot *Homo sapiens* database (18). Label-free quantitation with MaxQuant's standard-setting parameters was performed with the maximum of two miss cleavages, a mass tolerance of 0.6 D for the main search, trypsin as digesting enzyme, carbamidomethylation of cysteine as fixed modification, and the oxidation of methionine and acetylation of the protein N-terminus as variable modifications. Only peptides with a minimum of seven amino acids, as well as at least one unique peptide, were required for protein identification. Only proteins with at least two peptides, and at least one unique peptide, were considered as being identified and used for further data analysis. Protein false discovery rate (PFDR) was set at 1% and estimated by using the reversed search sequences. The maximal number of modifications per peptide was set to 5.

The MaxQuant txt file was loaded into Perseus (version 1.6.6), and max intensities and pairwise comparisons between conditions were done *via t*-tests (19). Missing values were also imputed in Perseus using a constant value (zero). The visualization and statistical analyses were conducted using the MultiExperiment Viewer (MeV) in the TM4 suite software (20). The final data containing protein name, accession number, peptide sequence, *Q*-value, and signal intensity were exported into an excel file.

Afterward, all protein data sets of three serum samples of each group were analyzed and only those commonly expressed among three samples in each group were selected. Then, those representative proteins in each group were analyzed for their intersection among the different sample groups using the Jvenn software (21). Finally, the proteins found in the sera of patients with CCA without LN metastasis, but not in other groups, were chosen as candidate proteins.

### Characterization of secretory proteins

In this study, three bioinformatics tools were used for protein selection. (I) the Plasma Proteome Database (PPD; Human Proteome Organization), which is one of the largest databases on plasma proteins (22). (II) SignalP software (version; Department of Bio and Health Informatics, Technical University of Denmark), which predicts classical secretory protein in mammalian sequences using a D-score >0.45 (23). (III) SecretomeP software (version 2.0; Department of Bio and Health Informatics, Technical University of Denmark), which predicts non-classical secretory protein using a Neural Network (NN) score >0.5 (24). In addition, to see the mRNA expression levels of the selected candidate protein in CCA (in GEPIA2, cholangiocarcinoma is CHOL) and normal tissues, we used the open-access database of Gene Expression Profiling Interactive Analysis 2 (GEPIA2; http://gepia2.cancer-pku.cn/; Peking University, Beijing, China) (25). *P*-value <0.05 was used as a statistically significant difference between CCA and HC groups.

### Western blot assay

Fifty micrograms of protein of the serum samples from HC, BBD, and CCA without LN metastasis were dissolved in sample buffer (10% sodium dodecylsulfate (SDS), 1M Tris-HCl, pH 6.8) and boiled for 5 min. The samples were loaded and separated in parallel with standard molecular weight markers on 12.5% SDS-PAGE at 120 V for 1 h at room temperature. After electrophoresis, proteins were electrically transferred onto PVDF membrane (GE Healthcare, Buckinghamshire, UK) for 1 h at room temperature. The membrane was blocked in 5% skimmed milk in Tris-buffered saline with 0.1% Tween-20 (1X TBS-T, pH 7.4) for 1 h at room temperature for non-specific protein blocking. The membrane was incubated with a rabbit polyclonal antibody against human PTPRS (1:2,000; Cat. No. orb630367; Biorbyt, Cambridge, UK) overnight at 4°C. The membrane was washed with 1X TBS-T and then incubated with a horseradish peroxidase-conjugated goat anti-rabbit IgG secondary antibody (1:5,000; Cat. No. ab7083; Abcam, Cambridge, UK) for 1 h at room temperature and washed with 1X TBS-T. The chemiluminescent signal was detected using enhanced chemiluminescence plus reagent (GE Healthcare, Buckinghamshire, UK) and quantified on Amersham imager 600 (GE Healthcare).

### Dot blot assay

In the preliminary study, the median and quartile deviation values of the relative intensity of PTPRS in 20 CCA and 20 HC were determined using dot blot assay. Then, the relative intensity of PTPRS from two groups was used for sample size calculation using the PS program (version 3.1.6) (26) and the minimum sample size necessary for comparison between CCA and HC was 55 samples.

A nitrocellulose membrane (GE Healthcare, Buckinghamshire, UK) was soaked in 1X TBS-T at room temperature and set on the Bio-Dot Microfiltration apparatus (Bio-Rad Laboratories, Inc.). Each serum sample was diluted to 1:2 with normal saline and pooled serum of 80 patients with CCA was used as a positive control for intensity normalization. Two microliters of each diluted sample were spotted onto the membrane. The membrane was soaked in 5% skimmed milk in 1X TBS-T for 1 h at room temperature for blocking non-specific binding. Then, the membrane was incubated with the rabbit polyclonal antibody against human PTPRS (1:2,000; Cat. No. orb630367; Biorbyt, Cambridge, UK) overnight at 4°C. The membrane was washed with 1X TBS-T and then incubated with horseradish peroxidase-conjugated goat anti-rabbit IgG secondary antibody (1:5,000; Cat. No. ab7083; Abcam, Cambridge, UK) for 1 h in room temperature and washed with 1X TBS-T. The chemiluminescent signal was detected using enhanced chemiluminescence plus reagent (GE Healthcare, Buckinghamshire, UK) and quantified by Amersham imager 600 (GE Healthcare). The signal intensity was measured using ImageJ software (ver. 1.52d; NIH, MD, USA). The experiment was performed in triplicate.

To prepare a standard curve, recombinant PTPRS protein with a known concentration (50 ng/ml) was serially two-fold diluted as 3.12, 6.25, 12.5, and 25 ng/ml. The intensities of PTPRS in the sera were normalized using PTPRS intensity in a positive control as relative intensity. Subsequently, the relative expression of PTPRS in each serum sample was calculated based on the standard curve prepared using the standard recombinant PTPRS protein.

### Sandwich enzyme-linked immunosorbent assay (ELISA)

Quantitative sandwich ELISA was performed according to the manufacturer's instruction (Cat. No. MBS930352; My Biosource, California, USA). Briefly, the plate was coated with a primary antibody specific to PTPRS. A total of 50 μl of standard, control, and samples were added to each well, and then the polyclonal antibody specific for PTPRS conjugated to horseradish peroxidase was added. The plate was incubated for 1 h. at 37°C. After washing, the substrate solution was added to each well for 15 min at 37°C and protect from light. The reaction was stopped and the absorbance was read on an ELISA reader using Multiskan GO (Thermo Fisher Scientific) at the optical density (OD) of 450 nm. The experiment was performed in triplicate and the results were calculated by reference to the standard curve.

### Statistical analysis

The data are presented as the median ± quartile deviation with the range (minimum to maximum) due to the obtained data being non-normal distribution. Comparisons between two groups and among overall groups were performed using the Mann-Whitney U test and the Kruskal-Wallis test. The association between clinical data and serum PTPRS level was performed using a chi-square test. A receiver operating characteristic (ROC) curve was performed, and the cut-off value was calculated using the Youden index (27). *P* in ROC was the probability that the observed sample area under the curve that predict the evidence of the test provided an ability to distinguish between two groups. Cumulative survival time was calculated using a Kaplan-Meier method and analyzed by log-rank test. The correlation coefficient between two variables was performed by Spearman's correlation test to detect collinearity. A correlation coefficient of <0.7 between two variables was indicated of no collinearity. And tolerance and variance inflation factor (VIF) values were performed to evaluate multicollinearity between variations, with tolerance >0.1 and VIF < 10 were indicated of no multicollinearity (28). The proportional hazard assumption was performed (29). The Cox proportional hazards regression model was used for univariate and multivariate analysis. GraphPad Prism software (version 8; GraphPad Software Inc.) and SPSS software (version 22; SPSS, Inc.) were used for statistical analysis. *P* < 0.05 was considered a statistically significant difference.

## Results

### Bioinformatic analysis to select secretory proteins

**Figure 1:**
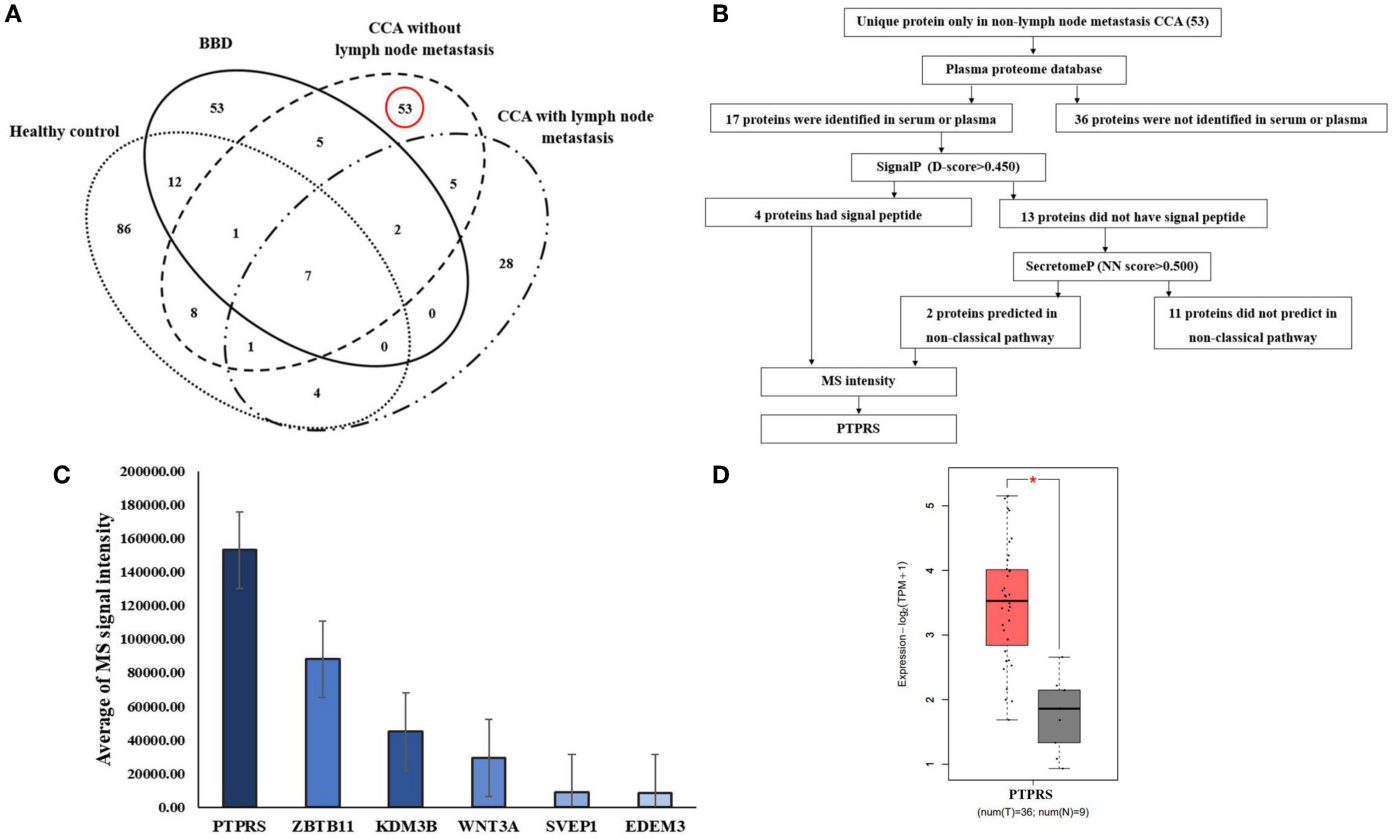
Identification of candidate proteins. **(A)** The Venn diagram presents the number of proteins in each group and the degree of overlapping of proteins. The dotted oval: total proteins identified in HC, the solid oval: total proteins identified in BBD group, the dashed oval: total proteins identified in CCA without LN metastasis, and the dash-dotted oval: total proteins identified in CCA with LN metastasis. **(B)** Flowchart of selection of secretory proteins in CCA without LN metastasis. **(C)** Average MS signal intensity level of six candidate proteins. **(D)** mRNA expression of PTPRS in CCA (as CHOL) tissue was analyzed by using GEPIA2. T, tumor (red box), N, normal (gray box). *Statistical significance (*P* < 0.05).

Using Venn diagram analysis as shown in Figure 1A, proteins uniquely expressed in the sera of CCA without LN metastasis group (*n* = 3) were selected (Supplementary Table 1) and their secretory protein nature was analyzed using bioinformatic tools of which flowchart is shown in Figure 1B. In CCA without the LN metastasis group, 82 proteins were identified in their sera. Among those 53 proteins were unique for this group, patients with CCA without LN metastasis. Then, using the plasma proteome database (PPD), 17 of those 53 proteins were identified to be present in the serum or plasma. Subsequently, using SignalP, four of 17 proteins were identified to have signal peptides and likely be secreted *via* a classical pathway. The rest of the 13 proteins were further analyzed using SecretomeP and two of them were identified as non-classical secretory proteins. Among four classical secretory proteins, lysine-specific demethylase 3B (KDM3B), protein Wnt-3a (WNT3A), protein tyrosine phosphatase receptor S (PTPRS), Sushi, von Willebrand factor type A, EGF, and pentraxin domain-containing protein 1 (SVEP1) and 2 non-classical secretory proteins, ER degradation-enhancing alpha-mannosidase-like protein (EDEM3) and Zinc finger and BTB domain-containing protein 11 (ZBTB11). We selected PTPRS as the biomarker candidate, due to the average of its MS signal intensity was the highest among six candidates as shown in Figure 1C and Supplementary Table 2. And the GEPIA2 database, the mRNA expression of PTPRS in CCA was significantly higher than in normal tissue (*P* < 0.05) (Figure 1D).

### Serum PTPRS levels of the CCA with/without LN metastasis, BBD, and healthy control groups

As a preliminary test, the specificity of the anti-PTPRS antibody to detect serum PTPRS protein in the sera of three each from CCA without LN metastasis, BBD, and healthy control (HC) groups was examined using Western blot analysis. As shown in Supplementary Figure 1, the antibody used in this study gave a single band of the expected molecular size of PTPRS for all nine samples examined (30).

**Figure 2:**
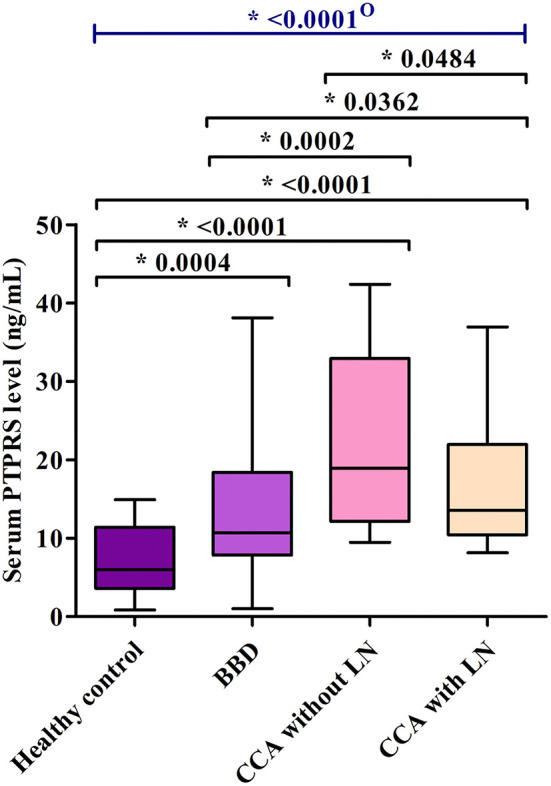
The comparison of serum PTPRS levels among healthy control group (*n* = 55), benign biliary disease (BBD) (*n* = 39), CCA without lymph-node metastasis (*n* = 38) and CCA with lymph-node metastasis (*n* = 38). The comparison was performed using the Mann Whitney U test and the Kruskal-Wallis test because the data distribution of PTPRS level was non-normal. A significant difference (*P* < 0.05): O, overall group.

Then, serum PTPRS levels of the bulk samples of CCA, BBD, and HC groups were measured semi-quantitatively using a dot blot assay system based on the standard curve created by using a standard PTPRS protein (Supplementary Figure 2). Representative dot blot membranes showing PTPRS in each serum sample are presented in Supplementary Figure 3. Serum samples were also shuffled and blotted on the membrane to ensure the reliability of the dot blot assay. As shown in Supplementary Figure 4, the data sets of ordinary and shuffled spot sheets gave a linear correlation with high reproducibility. The PTPRS levels in the sera of 38 with and 38 without LN metastasis, 39 patients with BBD, and 55 HC were presented in Figure 2. The median value of serum PTPRS level of the CCA group with/without LN metastasis was significantly higher than that of BBD or HC. The median serum PTPRS level of patients with CCA without LN metastasis was significantly higher than that of patients with CCA with LN metastasis. We also performed the correlation of serum PTPRS level between dot blot assay and ELISA and the data sets of dot blot and ELISA provided a linear correlation with high reproducibility (Supplementary Figure 5). The clinical characteristics of the participants of this study and CCA with unknown LN metastasis status were summarized in Table 1, Supplementary Table 3, respectively.

**Table 1:** The clinical characteristics of the participants of this study.

**Parameters**	**Healthy control**	**BBD**	**CCA**	**CCA without LN**	**CCA with LN**	
**(Normal range)**	**(*N* = 55)**	**(*N* = 39)**	**(*N* = 80)**	**metastasis (*N* = 38)**	**metastasis (*N* = 38)**	***P*-value**
Age	33 ± 6 (22–59)	59 ± 7 (33–76)	64 ± 5 (42–83)	63 ± 5 (45–76)	65 ± 5 (42–83)	<0.0001^a^, ^b^, ^c^, ^4^, ^e^
Total protein (6.5–8.8 g/dl)	NA	7.3 ± 0.4 (5.6–8.4)	7.4 ± 0.4 (4.6–9.1)	7.6 ± 0.3 (5.2–9.1)	7.3 ± 0.5 (4.6–9.1)	0.463
Albumin (3.8–5.4 g/dl)	NA	3.7 ± 0.6 (1.7–4.6)	4.0 ± 0.4 (2.4–5.0)	4.1 ± 0.4 (2.6–5.0)	4.0 ± 0.4 (2.4–4.8)	0.023^e^, ^f^, ^g^
Total bilirubin (0.25–1.5 mg/dl)	NA	2.6 ± 10.0 (0.2–56.4)	0.6 ±1.2 (0.2–16.8)	0.6 ± 1.2 (0.2–16.8)	0.7 ± 1.3 (0.2–6.4)	0.279
Direct bilirubin (0–0.5 mg/dl)	NA	0.7 ± 7.4 (0–33.2)	0.3 ± 1.1 (0.1–14.6)	0.3 ± 1.0 (0.1–14.6)	0.4 ± 1.1 (0.1–5.7)	0.858
ALT (4–36 U/L)	14 ± 4 (7–35)	36 ± 15 (10–145)	38 ± 20 (4–379)	35 ± 15 (4–257)	40 ± 19 (11–379)	<0.0001^a^, ^b^, ^c^, ^d^
AST (12–32 U/L)	20 ± 2 (14–28)	39 ± 20 (17–189)	43 ± 18 (14–598)	42 ± 12 (15–136)	44 ± 28 (14–598)	<0.0001^a^, ^b^, ^c^, ^d^
ALP (42–121 U/L)	50 ± 10 (26–81)	175 ± 136 (54–1,293)	177 ± 91 (35–1,068)	185 ± 81 (74–1,068)	167 ± 95 (35–712)	<0.0001^a^, ^b^, ^c^, ^d^
CEA (0–2.5 ng/ml)	NA	3.4 ± 3.0 (1.0–33.6)	5.2 ± 3.5 (1.0–1,000)	5.7 ± 3.2 (1.0–1,000)	4.4 ± 5.4 (1.2–1,000)	0.635
CA19–9 (0–37 U/ml)	NA	410 ± 478 (0.6–1,000)	120 ± 175 (0.6–1,000)	141 ± 311 (0.6–1,000)	240 ± 437 (0.6–1,000)	0.499
Survival time (days)	NA	554 ± 1,151 (17–5,592)	251 ± 118 (15–787)	237 ± 125 (15–672)	224 ± 128 (24–787)	0.167
Serum PTPRS level (ng/ml)	6.00 ± 3.73 (0.85–14.92)	10.70 ± 5.05 (1.01–38.09)	14.38 ± 9.42 (8.13–42.40)	19.69 ± 10.10 (9.48–42.40)	13.57 ± 5.38 (8.13–36.96)	<0.0001^a^, ^b^, ^c^, ^d^, ^e^, ^f^, ^g^, ^h^

Data are presented as the median ± quartile deviation and (minimum-maximum).

A significant difference between

(a) HC and BBD;

(b) HC and CCA;

(c) HC and CCA without LN metastasis;

(d) HC and CCA with LN metastasis;

(e) BBD and CCA;

(f) BBD and CCA without LN metastasis;

(g) BBD and CCA with LN metastasis;

(h) CCA without and with LN metastasis.

NA, not available; ALT, alanine transaminase; AST, aspartate transaminase; ALP, alkaline phosphatase; CEA, carcinoembryonic antigen; CA19-9, carbohydrate antigen 19-9. Note: Four of 80 CCA samples were lack of LN metastasis status.

### Evaluation of the diagnostic value of serum PTPRS level for CCA

**Figure 3:**
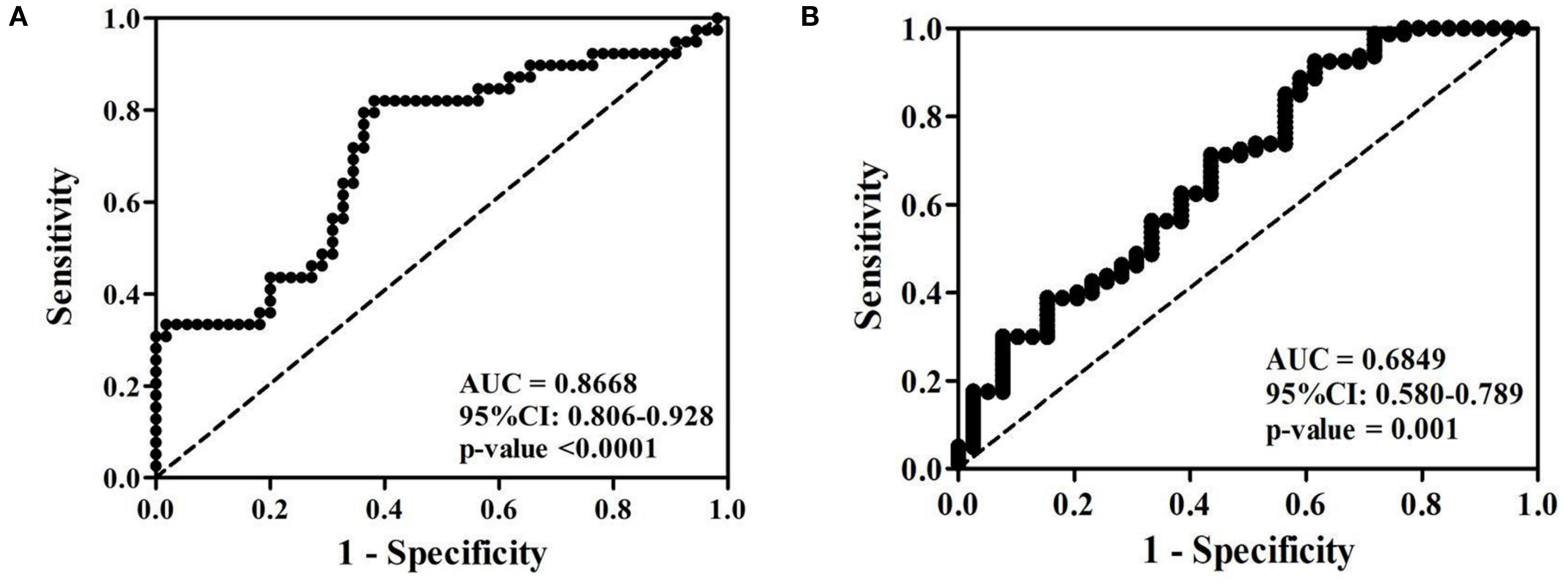
Receiver operating characteristic (ROC) curve of serum PTPRS level of patients with CCA against **(A)** healthy control and **(B)** BBD. *Statistically significant (*P* < 0.05).

To evaluate the diagnostic value of serum PTPRS level, receiver operating characteristic (ROC) curve analysis was performed. The cut-off of serum PTPRS level was calculated using the Youden index. The indexes were 6.22 and 9.24 ng/ml to distinguish CCA from HC (Figure 3A) and CCA from BBD (Figure 3B), respectively. The diagnostic test analysis, between CCA and HC groups, gave 97.5% sensitivity, 58.2% specificity, 77.7% positive predictive value (PPV), 100% negative predictive value (NPV), and 83% accuracy. Comparison between CCA and BBD group gave 92.5% sensitivity, 38.5% specificity, 75.5% PPV, 71.4% NPV, and 74.8% accuracy.

The sensitivity, specificity, and accuracy values of PTPRS were compared with these currently used serum markers, CEA, CA19-9, and ALP for distinguishing CCA from the BBD group. The diagnostic value of PTPRS had higher sensitivity, specificity, and accuracy which is presented in Table 2.

**Table 2:** The sensitivity, specificity, and accuracy values of currently used serum markers and PTPRS for distinguishing patients with CCA from those with BBD.

**Marker** ^a^	**Sensitivity (%)**	**Specificity (%)**	**Accuracy (%)**	**AUC (%)**	***P*-value**
PTPRS	92.5	38.5	74.8	0.6849	0.001^*^
CEA	81.8	26.7	63.9	0.6093	0.079
CA19-9	67.7	32.5	57.7	0.5682	0.179
ALP	75.0	30.9	64.7	0.5265	0.602

a Cut-off values: PTPRS 9.24 ng/ml, CEA 2.5 ng/ml, CA19-9 37 U/ml, ALP 121 U/L.

* Statistically significant (P < 0.05).

### Associations between serum PTPRS level and clinical parameters

The associations between serum PTPRS level and clinical parameters were examined to see the clinical importance of serum PTPRS level. For this purpose, patients with CCA were divided into high and low serum PTPRS levels using the median value (14.38 ng/ml), and the associations between serum PTPRS levels and clinical parameters were analyzed. The results are shown in Table 3. The serum PTPRS level was associated with albumin (*P* = 0.028), LN metastasis (*P* = 0.038) and survival time (*P* = 0.011) of patients with CCA, but not with other parameters.

**Table 3:** The associations between serum PTPRS level and clinical parameters.

**Clinical parameter**		**Serum PTPRS level**		
		**≤ 14.38 ng/ml (*n* = 40)**	**>14.38 ng/ml (*n* = 40)**	***P*-value**
Sex	Male (*n* = 57)	28 (35.0%)	29 (36.3%)	0.402
	Female (*n* = 23)	12 (15.0%)	11 (13.7%)	
Age		64 ± 5 (42–76)	64 ± 4 (45–83)	0.722
Lymph-node metastasis	No (*n* = 38)	14 (18.4%)	24 (31.6%)	0.038^*^
	Yes (*n* = 38)	23 (30.3%)	15 (19.7%)	
Total protein (g/dl)		7.3 ± 0.3 (4.7–8.3)	7.5 ± 0.6 (4.6–9.1)	0.115
Albumin (g/dl)		3.9 ± 0.6 (2.4–5.0)	4.1 ± 0.3 (2.4–4.8)	0.028^*^
Total bilirubin (mg/dl)		0.6 ± 1.2 (0.2–15.9)	0.6 ± 1.0 (0.2–16.8)	0.908
Direct bilirubin (mg/dl)		0.4 ± 1.2 (0.1–14.6)	0.3 ± 0.9 (0.1–13.7)	0.819
ALT (U/L)		41 ± 18 (4–379)	38 ± 21 (9–123)	0.784
AST (U/L)		40 ± 30 (14–598)	47 ± 16 (15–193)	0.481
ALP (U/L)		190 ± 115 (35–1,068)	157 ± 83 (68–665)	0.279
CEA (ng/ml)		4.7 ± 6.2 (1.0–728)	5.5 ± 2.5 (1.0–1,000)	0.507
CA19-9 (U/ml)		189 ± 380 (0.6–1,000)	169 ± 466 (0.6–1,000)	0.564
Survival time (days)		203.5 ± 223.9 (24–787)	345.0 ± 348.3 (15–742)	0.011^*^

* Statistically significant (P < 0.05). Value represents: Median ± Quartile deviation and (minimum-maximum). These variables were analyzed for the low and high levels of PTPRS (cut-off value at 14.38 ng/ml).

**Figure 4:**
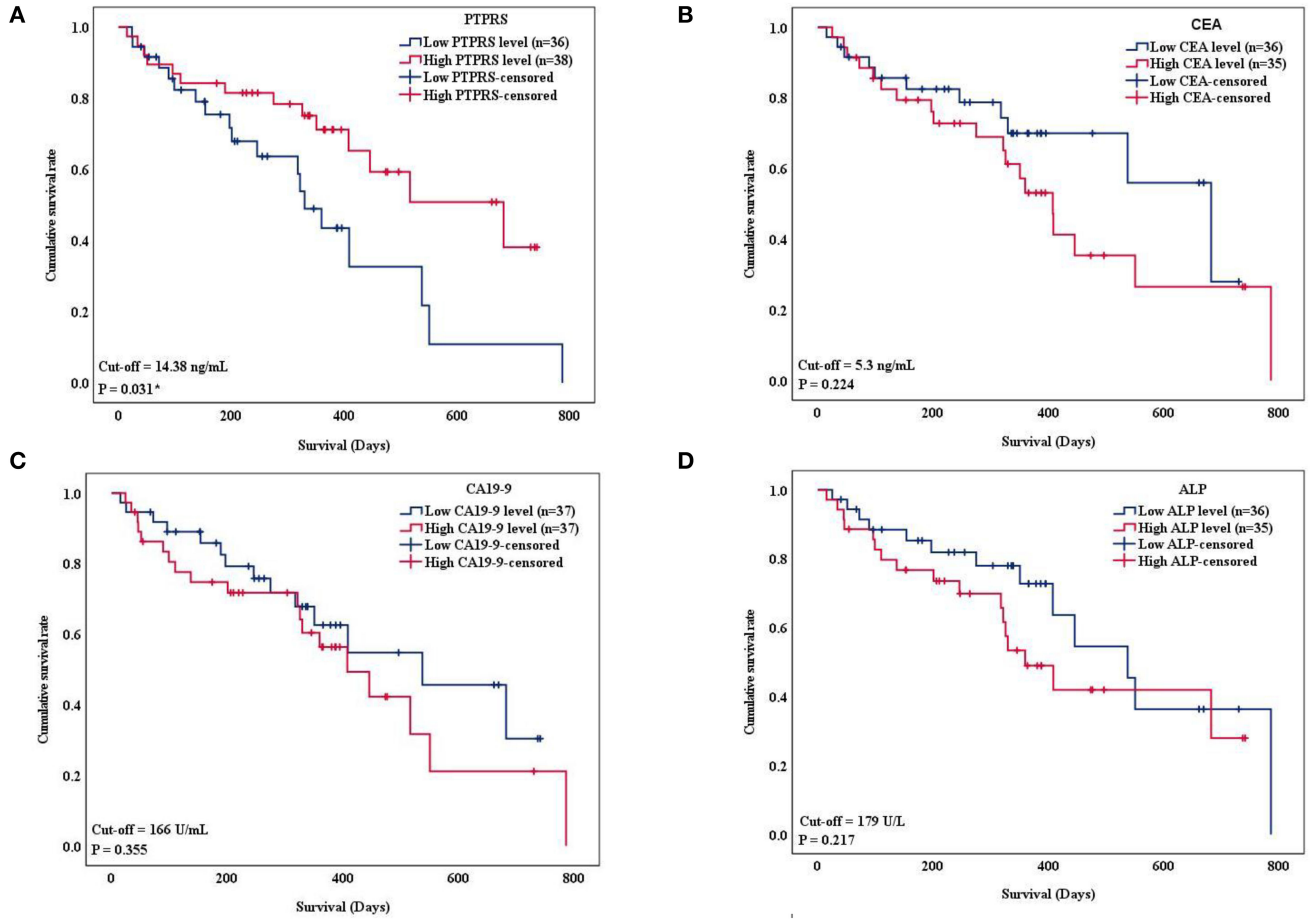
Kaplan-Meier curves show overall survival (OS) of patients with CCA based on the serum level of PTPRS, CEA CA19-9, and ALP, respectively. The curves showed OS of patients with CCA having a high level (red line) and low level (blue line) of the markers. The survival times were analyzed between high and low expression groups of **(A)** serum PTPRS level, **(B)** CEA level **(C)** CA19-9 level, and **(D)** ALP level (log-rank test; *P* = 0.031, 0.224, 0.355, and 0.217 respectively). * Statistically significant (*P* < 0.05).

Since serum PTPRS level was associated with the survival time of patients with CCA, the correlation between serum level of PTPRS, CEA, CA19-9, ALP, and the survival time of patients with CCA was analyzed using Kaplan-Meier analysis. Initially, the Spearman analysis was performed to examine the correlation between two variables (Supplementary Figure 6). The result showed that serum PTPRS level neither correlated with CEA, CA19-9 nor ALP. Because the distribution of PTPRS data of patients with CCA was non-normally distributed, we used the median value as a cut-off for the CCA group. The survival time of patients with CCA was significantly (*P* = 0.031 by log-rank test) longer in the high serum PTPRS level group than in the low serum PTPRS level group (Figure 4A). In contrast, the survival time of patients with CCA was not significantly different (*P* = 0.224, *P* = 0.355, and *P* = 0.217 respectively by log-rank test) between high and low serum levels of CEA, CA19-9, and ALP (Figures 4B–D).

The proportional hazard assumption was performed. And the time-dependent covariate was not significant (*P* > 0.05), and the proportional hazard assumption was met. The multivariate Cox regression analysis demonstrated that only the serum PTPRS level was an independent prognostic marker for CCA with a hazard ratio of 0.37 (95% CI, 0.17–0.81) with *P* = 0.013 as presented in Table 4. The correlation coefficient between every variable was <0.7 (Supplementary Table 4), and the tolerance and VIF values of variables were >0.1 and < 10, respectively (Supplementary Table 5), indicating no collinearity between the variables.

**Table 4:** The Cox proportional hazards regression analysis of clinicopathological parameters and the serum PTPRS level of patients with CCA.

**Clinical parameter**	**Univariate analysis**		**Multivariate analysis**	
	**HR (95% CI)**	***P*-value**	**HR (95% CI)**	***P*-value**
Gender (Female or male)	1.16 (0.78–1.72)	0.461	0.95 (0.40–2.28)	0.915
Age (≤ 60 or >60 yr)	1.42 (0.65–3.10)	0.381	1.62 (0.66–3.99)	0.298
Total protein (≤ 8.8 or >8.8 g/dl)	3.89 (0.91–16.62)	0.066	9.33 (1.51–57.56)	0.056
Total bilirubin (≤ 1.5 or > 1.5 mg/dl)	1.41 (0.66–3.01)	0.317	2.20 (0.30–18.76)	0.470
Direct bilirubin (≤ 0.5 or >0.5 mg/dl)	1.18 (0.57–2.44)	0.661	0.53 (0.07–4.13)	0.541
ALT (≤ 36 or >36 U/L)	0.75 (0.37–1.53)	0.426	0.76 (0.29–2.03)	0.586
AST (≤ 32 or >32 U/L)	0.95 (0.46–1.98)	0.889	1.17 (0.44–3.13)	0.753
ALP (≤ 121 or >121 U/L)	1.02 (0.46–2.28)	0.957	1.23 (0.47–3.24)	0.674
CEA (≤ 2.5 or >2.5 ng/ml)	1.15 (0.44–3.01)	0.778	1.07 (0.41–2.83)	0.899
CA19-9 (≤ 37 or >37 U/ml)	1.19 (0.49–2.90)	0.706	1.39 (0.53–3.66)	0.508
Serum PTPRS level (≤ 14.38 or > 14.38 ng/ml)	0.47 (0.23–0.95)	0.035^*^	0.37 (0.17–0.81)	0.013^*^

* Statistically significant (P < 0.05).

HR, Hazard ratio, CI, confidence interval.

## Discussion

In this study, we used proteomic and three bioinformatics tools, SignalP, SecretomeP, and PPD, to select candidate proteins of secretory protein nature for the diagnosis/prognosis of CCA. Bioinformatic analysis suggested that PTPRS was predicted to be a secretory protein in the conventional secretory pathway, which as analyzed by SignalP 5, is listed in PPD with the highest expression of mass spectrometry for validation. Then, serum PTPRS level of patients with CCA, patients with BBD, and HC was semi-quantitatively measured using a dot-blot assay and their diagnostic values were examined. The results showed that the serum PTPRS level of patients with CCA was significantly higher than that of BBD and HC groups. In patients with CCA, serum PTPRS level was associated with serum albumin, LN metastasis, and survival time of patients. Moreover, serum PTPRS level was found to be an independent prognostic marker in CCA.

Protein tyrosine phosphatase receptor S is a member of the class I protein tyrosine phosphatase (PTP) family and its gene is located on chromosome 19p13.3 (31). PTPRS plays important physiological roles in neurogenesis, spinal cord injury and repair, and autophagy (32, 33). PTPRS is expressed in multiple immune cells, including plasmacytoid dendritic cells (34, 35), and is a negative regulator for the development of autoimmune encephalomyelitis (36). PTPRS was reported also as a tumor suppressor in several cancers (37–41), and also the deletion or mutation of PTPRS was detected in several types of cancer such as colorectal cancer (40), head and neck squamous-cell carcinoma (37), and CCA (42, 43). The mutation type of PTPRS in CCA was missense and the frequency of mutation rate was 3.2% (42). Nevertheless, it may be because PTPRS is primarily a signal molecule, its expression in the sera of patients with CCA is lacking. To our best knowledge, the present results are the first report of serum PTPRS levels of patients with CCA, patients with BBD, and HC. We also found that the high serum PTPRS level was associated with high serum albumin, non-LN metastasis, and the long survival time of patients with CCA. The previous studies demonstrated that OS was longer in patients with CCA with high serum albumin compared with that of patients with CCA with low albumin, suggesting that albumin could be a prognostic marker for CCA (44, 45). The low PTPRS expression was associated with LN metastasis, which was similar to hepatocellular carcinoma (HCC) (38). The possible role of PTPRS in tumor progression was studied in several types of cancer. In HCC, PTPRS is act as a metastasis suppressor, and loss of PTPRS can increase the activity of epidermal growth factor receptor (EGFR) signaling and promote the epithelial-mesenchymal transition (EMT) process in metastasis (38). Also, in HCC, PTPRS could dephosphorylate and interact with STAT3. PTPRS-STAT3 axis mediated in tumor suppressor function of bone morphologic protein-10 (BMP-10) (46). In head and neck cancer, loss of PTPRS promoted EGFR activity in the EGFR/phosphoinositide 3 kinase (PI3K) pathway (37). In colorectal cancer, PTPRS negatively regulates the RAS pathway, so the decrease of PTPRS in cell lines increased the ERK activity (39).

In terms of the diagnostic value of PTPRS, ROC analysis provided sensitivity, specificity, PPV, NPV, and accuracy of 92.5%, 38.5%, 75.5%, 71.4% and 74.8%, respectively, with a cut-off value of 9.24 ng/ml. In this study, we compared the diagnostic value between serum PTPRS and currently used markers including CEA, CA19-9, and ALP that have been applied in routine clinical (6, 47). The diagnostic value of serum PTPRS was found to be better than currently used markers. Especially, these currently used markers could not differentiate between patients with CCA and BBD.

In this study, the OS of patients with CCA was found to be longer in the patients with high serum PTPRS levels than in those with low levels. In addition, PTPRS was identified as an independent prognostic marker and superior to CA19-9, CEA, or ALP, which are currently applied prognostic markers in clinical practice (48). Related to our results, an association of high expression of PTPRS in tumor tissues with the longer OS of patients has been reported in HCC (38), head and neck cancer (37), and malignant peripheral nerve sheath tumor (49). Also, in HCC, high PTPRS expression in the tumor was associated with non-aggressive tumor characteristics and a low risk of postoperative recurrence (38).

In conclusion, the high serum PTPRS level was associated with high albumin level, non-LN metastasis, and favorable overall survival of the patients with CCA. PTPRS was identified as an independent prognostic marker for CCA superior to CA19-9, CEA, and ALP. The limitation of this study is lacking some critical information about patients such as the tumor-node-metastasis staging, tumor burden, and post-surgical treatment for biomarker evaluation. Diagnostic and prognostic value of serum PTPRS levels should be investigated further in other cancer patients. The biological roles of PTPRS in the progression and development of CCA still remain unclear. Therefore, the possible roles of PTPRS in CCA cells will be investigated in the future using gene transfection/silencing.

## Data availability statement

The original contributions presented in the study are included in the article/Supplementary material, further inquiries can be directed to the corresponding author/s.

## Author contributions

AS, PT, TP, SR, and SP provided the concept for the research. ML and SP designed the study. SR performed the software analysis. ML performed the experiment and original draft preparation. TP and SP edited the manuscript. All authors have read and agreed to the published version of the manuscript.

## Funding

This research was funded by the research fund for supporting the lecturer to admit high potential student to study and research on his expert program year 2019, Graduate school, Khon Kaen University, Thailand (Grant No. 621H116).

## Conflict of interest

The authors declare that the research was conducted in the absence of any commercial or financial relationships that could be construed as a potential conflict of interest. The reviewer JC declared a shared affiliation with the authors ML, AS, PT, TP, and SP to the handling editor at the time of review.

## Publisher's note

All claims expressed in this article are solely those of the authors and do not necessarily represent those of their affiliated organizations, or those of the publisher, the editors and the reviewers. Any product that may be evaluated in this article, or claim that may be made by its manufacturer, is not guaranteed or endorsed by the publisher.

## Supplementary material

Supplementary Figure 1 The expression of PTPRS in serum samples were investigated by western blot.

Supplementary Figure 2 PTPRS standard curve for dot blot assay.

Supplementary Figure 3 Representative dot blot membranes of PTPRS in serum samples obtained from the Amersham imager 600 analyzer.

Supplementary Figure 4 Shuffling of serum samples.

Supplementary Figure 5 The correlation of serum PTPRS level between dot blot assay and ELISA.

Supplementary Figure 6 The Spearman's correlation tested between the levels of PTPRS and CEA, CA19-9 or ALP.

Supplementary Table 1 The MS signal intensity level of common 53 unique proteins found in 3 CCA without LN metastasis serum samples.

Supplementary Table 2 The MS signal intensity level of six candidate proteins in CCA without LN metastasis group.

Supplementary Table 3 The clinical characteristics of CCA with unknown LN metastasis status.

Supplementary Table 4 The correlation coefficient between variables.

Supplementary Table 5 The tolerance and variance inflation factor (VIF) values of each variable.
