# Supplementary figures and images for: High expression of protein tyrosine phosphatase receptor S (PTPRS) is an independent prognostic marker for cholangiocarcinoma
Source: Front Public Health. 2022 Aug 1;10:835914. doi: 10.3389/fpubh.2022.835914 (PMC9387352; doi:10.3389/fpubh.2022.835914)

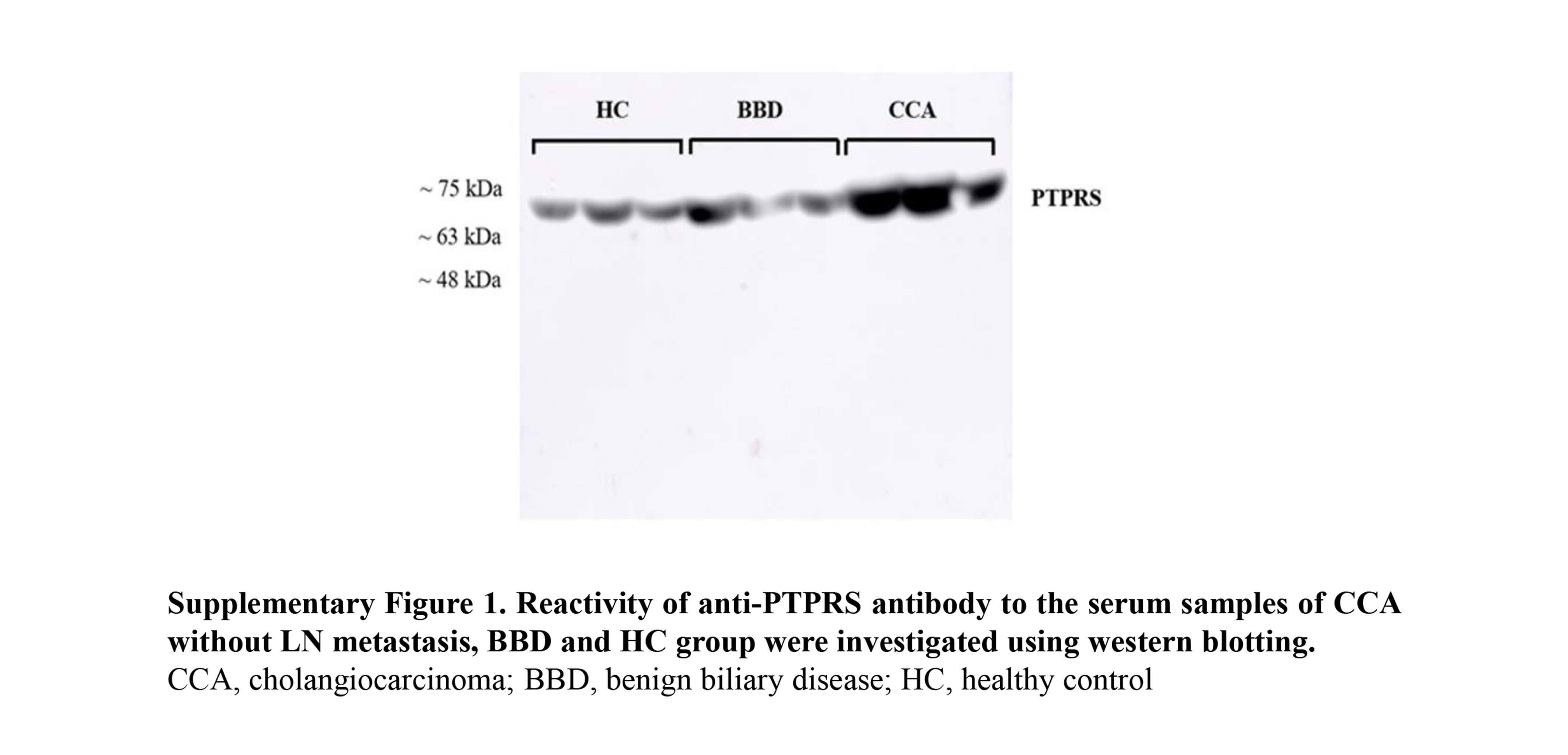

Supplement: Supplementary Figure 1 — The expression of PTPRS in serum samples were investigated by western blot. [file Image_1.TIF]

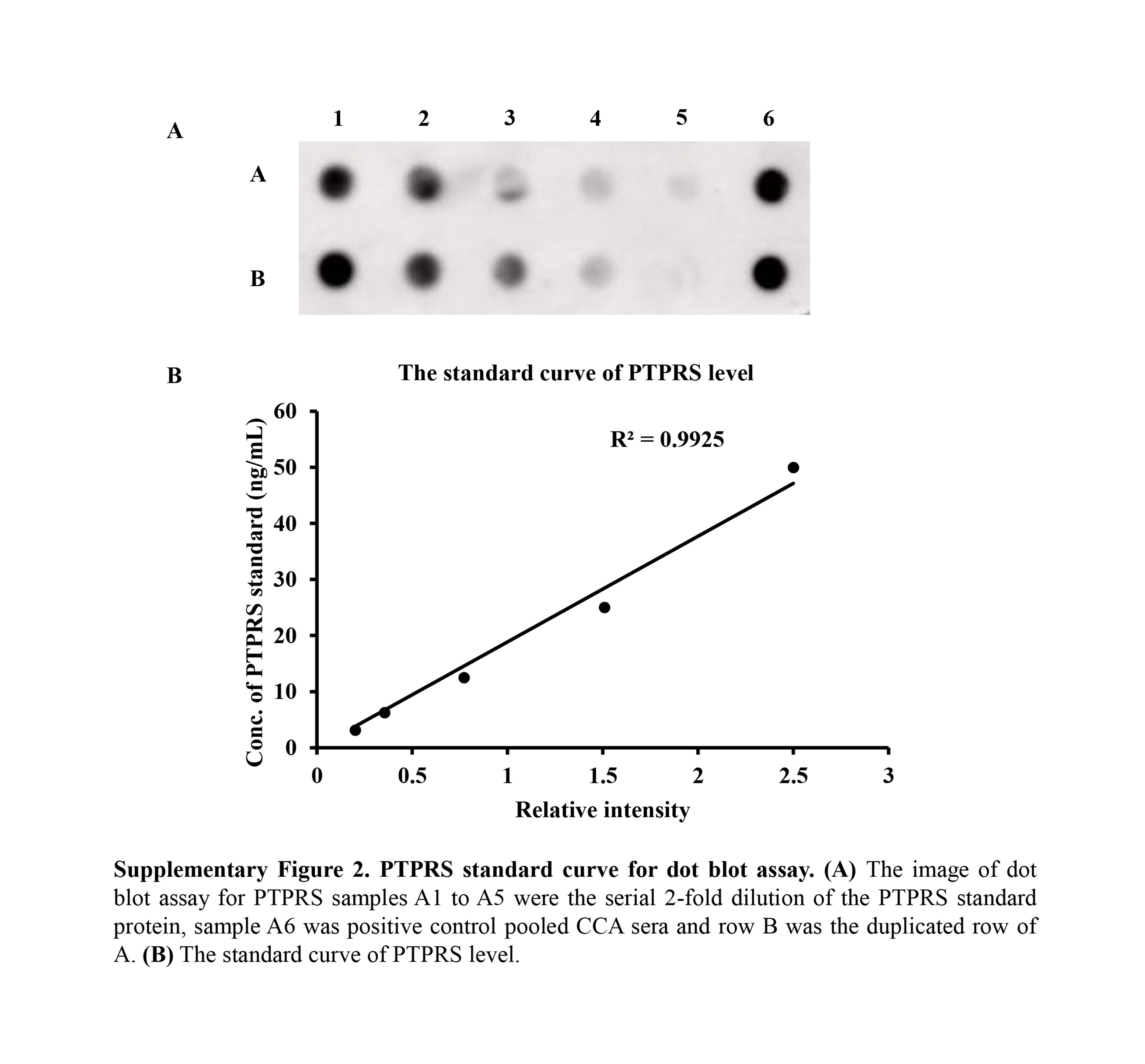

Supplement: Supplementary Figure 2 — PTPRS standard curve for dot blot assay. [file Image_2.TIF]

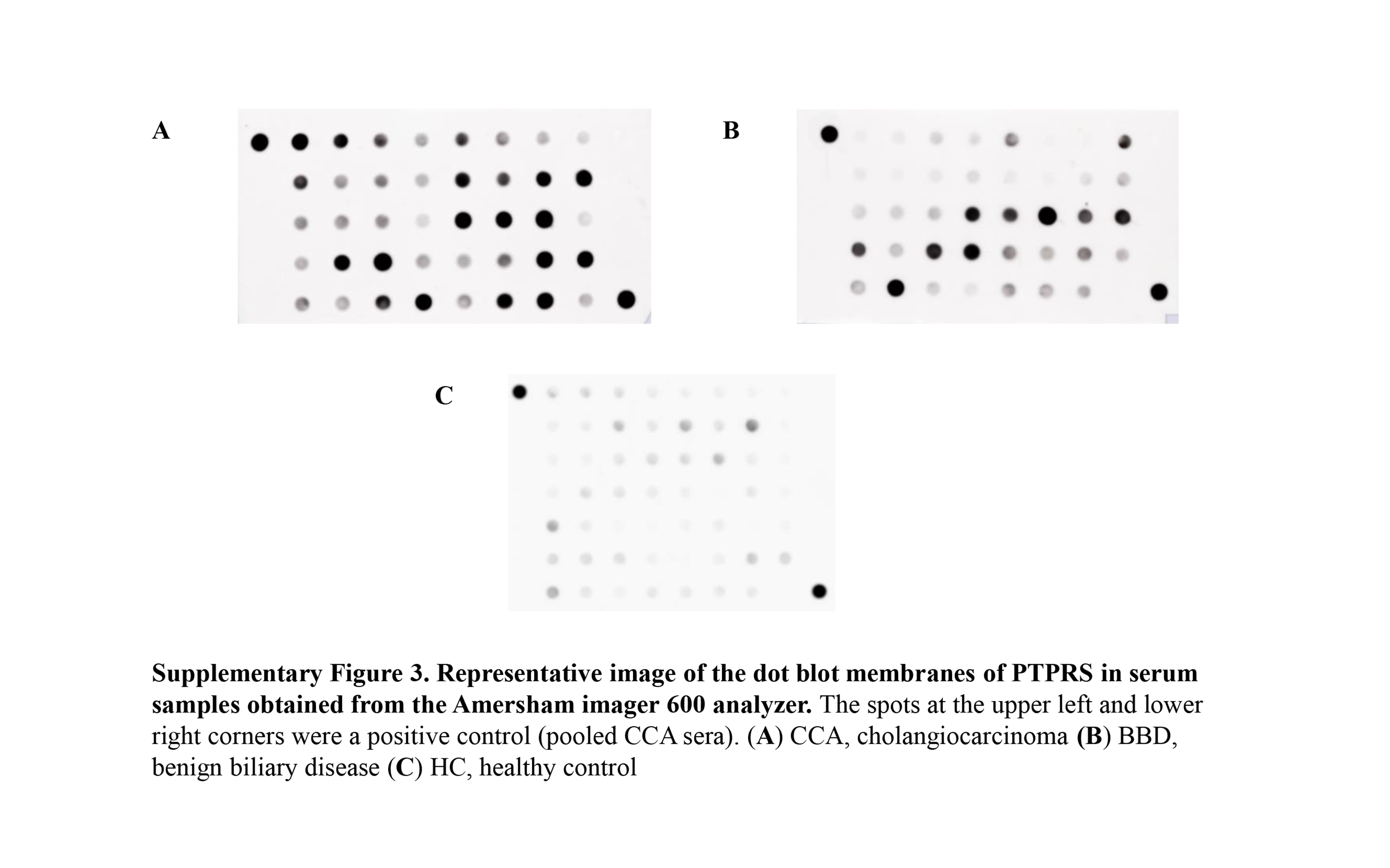

Supplement: Supplementary Figure 3 — Representative dot blot membranes of PTPRS in serum samples obtained from the Amersham imager 600 analyzer. [file Image_3.TIF]

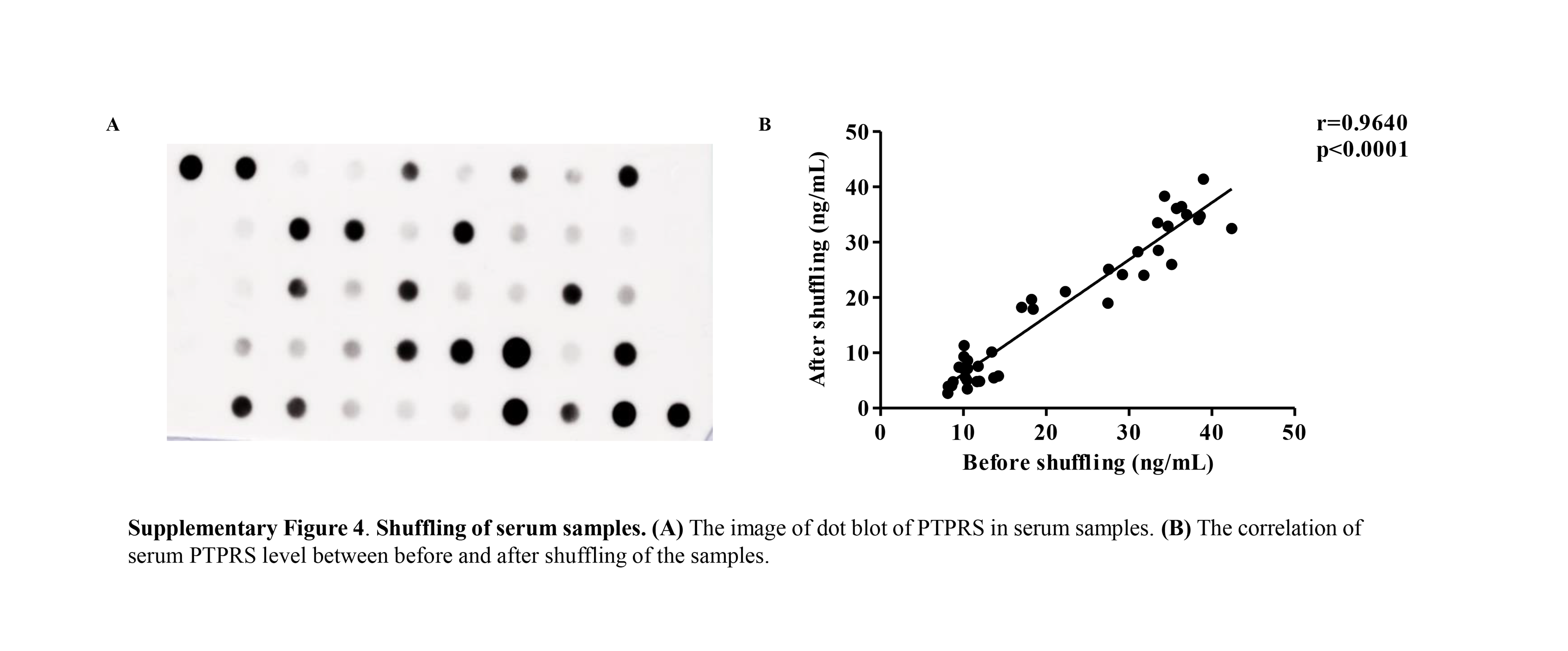

Supplement: Supplementary Figure 4 — Shuffling of serum samples. [file Image_4.TIF]

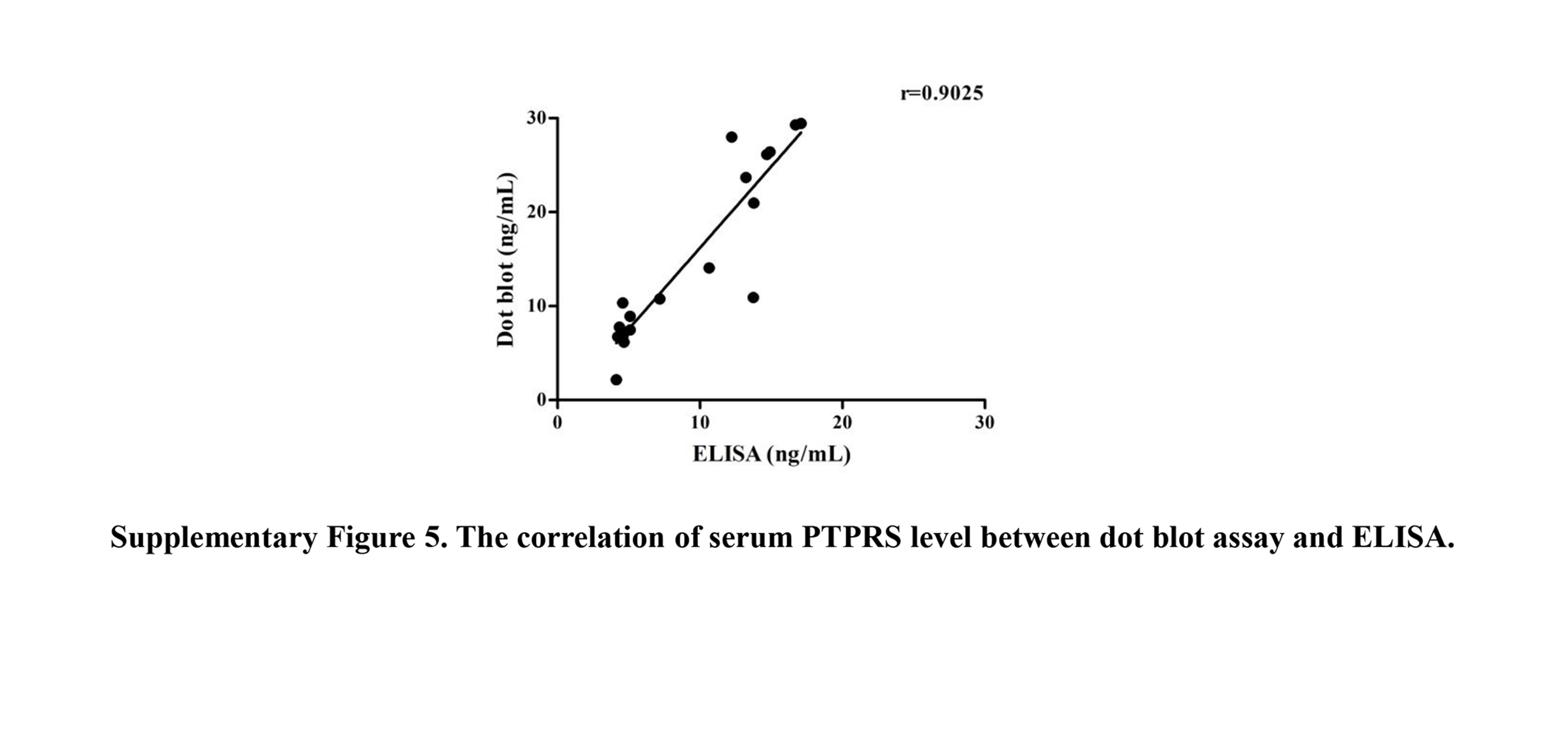

Supplement: Supplementary Figure 5 — The correlation of serum PTPRS level between dot blot assay and ELISA. [file Image_5.TIF]

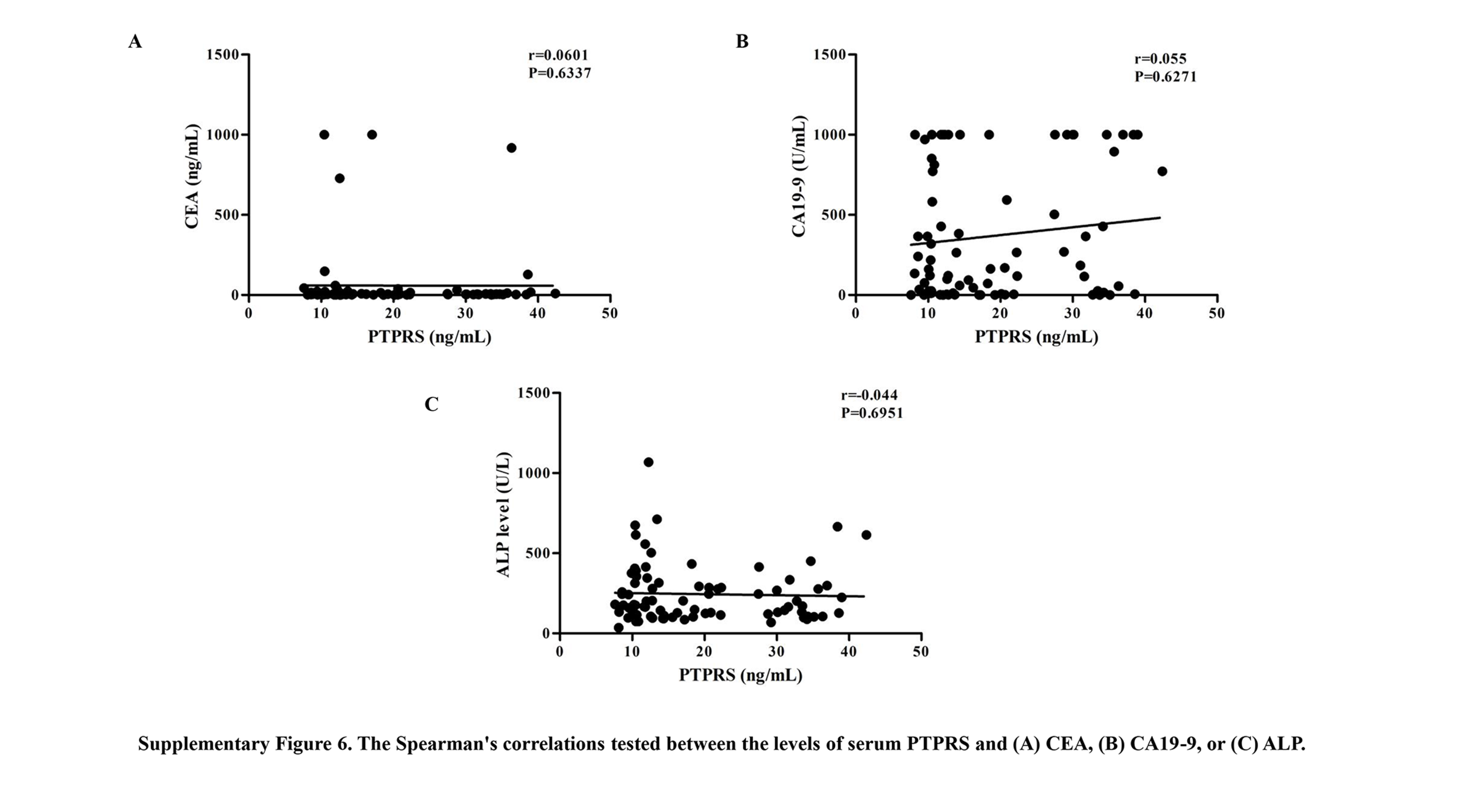

Supplement: Supplementary Figure 6 — The Spearman's correlation tested between the levels of PTPRS and CEA, CA19-9 or ALP. [file Image_6.TIF]
