# Supplementary material for: High expression of protein tyrosine phosphatase receptor S (PTPRS) is an independent prognostic marker for cholangiocarcinoma
Source: Front Public Health. 2022 Aug 1;10:835914. doi: 10.3389/fpubh.2022.835914 (PMC9387352; doi:10.3389/fpubh.2022.835914)
Supplement: Supplementary Table 1 — The MS signal intensity level of common 53 unique proteins found in 3 CCA without LN metastasis serum samples. [file Table_1.DOCX]

Supplementary Material

**Supplementary Table 1**. The MS signal intensity level of common 53 unique proteins found in 3 CCA without LN metastasis serum samples.

| **Protein name** | **Accession number** | **N1** | **N2** | **N3** | **Average of MS** |
| --- | --- | --- | --- | --- | --- |
| Apolipoprotein B (Including Ag(X) antigen) | C0JYY2 | 13937 | 24705 | 1346609 | 461750 |
| CDK5 regulatory subunit-associated protein 2 | A0A0A0MRG9 | 148922 | 10228 | 70660 | 76603 |
| Coiled-coil domain-containing protein 177 | Q9NQR7 | 7441 | 7847 | 9555 | 8281 |
| Crooked neck-like protein 1 | Q9BZJ0 | 13878 | 6614 | 576755 | 199082 |
| Cytochrome P450, family 4, subfamily F, polypeptide 3, isoform CRA_b | A0A024R7I2 | 6534 | 26716 | 1671834 | 568361 |
| Delta isoform of regulatory subunit B56, protein | Q59EF0 | 59959 | 2535 | 996079 | 352858 |
| Digestive organ expansion factor homolog | Q68CQ4 | 7740 | 9701 | 64647 | 27363 |
| ELKS/Rab6-interacting/CAST family member 1 | G8JLD3 | 193987 | 2913 | 165263 | 120721 |
| Endoribonuclease Dicer | Q9UPY3 | 52696 | 12238 | 150145 | 71693 |
| ER degradation-enhancing alpha-mannosidase-like protein 3 | Q9BZQ6 | 3412 | 16657 | 5857 | 8642 |
| Eukaryotic translation initiation factor 3 subunit A | J9R021 | 20490 | 1917772 | 27038 | 655100 |
| Eukaryotic translation initiation factor 3 subunit C | B4DRU0 | 25070 | 41099 | 197953 | 88041 |
| F-actin monooxygenase | A0A2R8YFA9 | 113042 | 232424 | 53309 | 132925 |
| FBLN7 protein | Q8IY13 | 36586 | 167709 | 62688 | 88994 |
| GNAS complex locus, isoform CRA_c | H0UI78 | 14235 | 21417 | 96190 | 43948 |
| Laminin subunit alpha-2 | A0A087WX80 | 43647 | 772781 | 29170 | 281866 |
| Lymphocyte cytosolic protein 2 | A0A0D9SGG1 | 11096 | 10892 | 17251 | 13080 |
| Lysine-specific demethylase 3B | Q7LBC6 | 9166 | 7172 | 119033 | 45123 |
| Meiosis-specific nuclear structural protein 1 | Q8NEH6 | 13711 | 9770 | 10172 | 11217 |
| Methyltransferase TARBP1 | Q13395 | 29071 | 22780 | 3295806 | 1115886 |
| Myosin heavy chain 11 smooth muscle isoform | B1PS43 | 9441 | 22059 | 348935 | 126812 |
| Myotubularin-related protein 5 | O95248 | 108182 | 17864 | 151672 | 92573 |
| Nuclear receptor co-repressor 1, isoform CRA_a | A0A024RD47 | 42770 | 6716 | 19591 | 23025 |
| Nucleolar complex protein 4 homolog | Q9BVI4 | 5458 | 19860 | 226006 | 83775 |
| Putative p150 | O00360 | 189114 | 9930 | 43963 | 81003 |
| PH-interacting protein | Q8WWQ0 | 8828 | 11558 | 31022 | 17136 |
| Phosphatidylinositol-4,5-bisphosphate 3-kinase | A0A024R720 | 144109 | 70167 | 25505 | 79927 |
| PLA2R1 protein | B7ZML4 | 110119 | 27513 | 43744 | 60458 |
| Protein FAM184B | Q9ULE4 | 344418 | 10712 | 2777 | 119302 |
| Protein KIBRA | H3BLZ3 | 19429 | 10457 | 91374 | 40420 |
| Protein shortage in chiasmata 1 ortholog | A6PVK7 | 7671 | 20602 | 37394 | 21889 |
| Protein unc-13 homolog A | F8W059 | 11184 | 13471 | 121829 | 48828 |

**Supplementary Table 1**. The MS signal intensity level of common 53 unique proteins found in 3 CCA without LN metastasis serum samples. (cont.)

| **Protein name** | **Accession number** | **N1** | **N2** | **N3** | **Average of MS** |
| --- | --- | --- | --- | --- | --- |
| Protein Wnt-3a | P56704 | 14603 | 61402 | 12109 | 29371 |
| Protocadherin Fat 1 | A0A087WVP1 | 10939 | 14640 | 36110 | 20563 |
| Protein tyrosine phosphatase receptor S | Q13332 | 27271 | 75873 | 355848 | 152997 |
| Ribonuclease 3 | Q9NRR4 | 112292 | 26461 | 64441 | 67732 |
| Probable ribonuclease ZC3H12C | Q9C0D7 | 9431 | 26890 | 40965 | 25762 |
| Serine hydroxymethyltransferase | B4DJQ3 | 14063 | 325478 | 7324 | 115622 |
| Serine/threonine-protein kinase Nek1 | Q96PY6 | 3538 | 111609 | 1717406 | 610851 |
| Sushi, von Willebrand factor type A, EGF and pentraxin domain-containing protein 1 | Q4LDE5 | 3090 | 1897 | 21236 | 8741 |
| Talin-2 | Q9Y4G6 | 668098 | 45970 | 201553 | 305207 |
| Telomerase reverse transcriptase | O14746 | 12148 | 66682 | 137913 | 72247 |
| Testis-expressed protein 15 | A0A1W2PS94 | 80194 | 2024 | 177419 | 86546 |
| Tetratricopeptide repeat protein 6 | G3V3A5 | 1246196 | 367988 | 4699904 | 2104696 |
| Transactivator | A0A126LAY8 | 2535 | 8839 | 7678 | 6350 |
| Transient receptor potential cation channel subfamily M | Q9BX84 | 16578 | 789840 | 151064 | 319161 |
| Tyrosine-protein phosphatase non-receptor type 13 | Q12923 | 13706 | 14787 | 162266 | 63586 |
| Ubiquitin specific protease 42 | A4D2N6 | 62341 | 115178 | 42770 | 73429 |
| Uncharacterized protein DKFZp686A111 | Q6AHZ7 | 438586 | 14787 | 984956 | 479443 |
| Zinc finger and BTB domain-containing protein 11 | O95625 | 20008 | 4368 | 239788 | 88055 |
| Zinc finger DBF-type containing 2 version 1 | N0DVB2 | 1570508 | 1196260 | 478746 | 1081838 |
| Zinc finger protein 749 | O43361 | 43385 | 16722 | 40536 | 33548 |
| Zinc finger protein 808 | Q8N4W9 | 59578 | 38570 | 74420 | 57523 |

N, the MS intensity of protein in CCA without LN metastasis serum samples.
