# Supplementary material for: High expression of protein tyrosine phosphatase receptor S (PTPRS) is an independent prognostic marker for cholangiocarcinoma
Source: Front Public Health. 2022 Aug 1;10:835914. doi: 10.3389/fpubh.2022.835914 (PMC9387352; doi:10.3389/fpubh.2022.835914)
Supplement: Supplementary Table 2 — The MS signal intensity level of six candidate proteins in CCA without LN metastasis group. [file Table_2.DOCX]

Supplementary Material

**Supplementary** **Table 2**. The MS signal intensity level of 6 candidate proteins in CCA without LN metastasis group.

| **Protein name** | **Accession number** | **Average of MS intensity** |
| --- | --- | --- |
| Protein tyrosine phosphatase receptor S | Q13332 | 152997 |
| Zinc finger and BTB domain-containing protein 11 | O95625 | 88055 |
| Lysine-specific demethylase 3B | Q7LBC6 | 45123 |
| Protein Wnt-3a | P56704 | 29371 |
| Sushi, von Willebrand factor type A, EGF and pentraxin domain-containing protein 1 | Q4LDE5 | 8741 |
| ER degradation-enhancing alpha-mannosidase-like protein 3 | Q9BZQ6 | 8642 |
