# Supplementary material for: High expression of protein tyrosine phosphatase receptor S (PTPRS) is an independent prognostic marker for cholangiocarcinoma
Source: Front Public Health. 2022 Aug 1;10:835914. doi: 10.3389/fpubh.2022.835914 (PMC9387352; doi:10.3389/fpubh.2022.835914)
Supplement: Supplementary Table 3 — The clinical characteristics of CCA with unknown LN metastasis status. [file Table_3.DOCX]

Supplementary Material

**Supplementary** **Table 3**. The clinical characteristics of CCA with unknown LN metastasis status.

| Parameter (Normal range) | CCA with unknown lymph node metastasis group (N=4) |
| --- | --- |
| Age | 63 ± 2 (57 - 68) |
| Total protein (6.5 - 8.8 g/dL) | 7.9 ± 0.6 (6.1 - 8.5) |
| Albumin (3.8 -5.4 g/dL) | 4.1 ± 0.2 (3.1 - 5.0) |
| Total bilirubin (0.25 - 1.5 mg/dL) | 0.5 ± 0.1 (0.3 - 9.7) |
| Direct bilirubin (0 - 0.5 mg/dL) | 0.2 ± 0.0 (0.1 - 8.3) |
| ALT (4 - 36 U/L) | 37 ± 12 (15 - 111) |
| AST (12 - 32 U/L) | 38 ± 8 (29 - 132) |
| ALP (42 - 121 U/L) | 186 ± 65 (74 - 665) |
| CEA (0 - 2.5 ng/mL) | 72.1 ± 58.1 (2.6 - 148.6) |
| CA19-9 (0 - 37 U/mL) | 589 ± 268 (5.3 - 1000) |
| Survival time (days) | 273 ± 24 (201 - 330) |
| Serum PTPRS level (ng/mL) | 9.72 ± 4.70 (8.62 - 38.39) |

Data are presented as the median ± quartile deviation and (minimum - maximum). ALT, alanine transaminase; AST, aspartate transaminase; ALP, alkaline phosphatase; CEA, carcinoembryonic antigen; CA19-9, carbohydrate antigen 19-9.
