# Supplementary material for: High expression of protein tyrosine phosphatase receptor S (PTPRS) is an independent prognostic marker for cholangiocarcinoma
Source: Front Public Health. 2022 Aug 1;10:835914. doi: 10.3389/fpubh.2022.835914 (PMC9387352; doi:10.3389/fpubh.2022.835914)
Supplement: Supplementary Table 4 — The correlation coefficient between variables. [file Table_4.DOCX]

Supplementary Material

**Supplementary** **Table 4**. The correlation coefficient between measured variables.

|  | Age | Total protein | Total bilirubin | Direct bilirubin | ALT | AST | ALP | CEA | CA19-9 |
| --- | --- | --- | --- | --- | --- | --- | --- | --- | --- |
| Total protein | -0.031 |  |  |  |  |  |  |  |  |
| Total bilirubin | 0.205 | -0.113 |  |  |  |  |  |  |  |
| Direct bilirubin | 0.175 | -0.185 | 0.614 |  |  |  |  |  |  |
| ALT | -0.082 | -0.175 | 0.370 | 0.129 |  |  |  |  |  |
| AST | -0.043 | -0.224 | 0.398 | 0.472 | 0.554 |  |  |  |  |
| ALP | -0.046 | 0.080 | 0.262 | 0.356 | 0.239 | 0.288 |  |  |  |
| CEA | -0.004 | 0.088 | 0.181 | 0.173 | 0.120 | 0.188 | -0.117 |  |  |
| CA19-9 | 0.024 | 0.089 | 0.088 | 0.258 | 0.122 | 0.289 | 0.245 | -0.0004 |  |
| PTPRS | -0.051 | 0.159 | -0.026 | -0.152 | -0.070 | -0.027 | -0.044 | 0.0601 | 0.055 |
