# Supplementary material for: High expression of protein tyrosine phosphatase receptor S (PTPRS) is an independent prognostic marker for cholangiocarcinoma
Source: Front Public Health. 2022 Aug 1;10:835914. doi: 10.3389/fpubh.2022.835914 (PMC9387352; doi:10.3389/fpubh.2022.835914)
Supplement: Supplementary Table 5 — The tolerance and variance inflation factor (VIF) values of each variable. [file Table_5.DOCX]

Supplementary Material

**Supplementary** **Table 5**. The tolerance and variance inflation factor (VIF) values of variable. PTPRS was regarded as the dependent variable and the remaining factors were independent variables.

|  |  | Age | Total protein | Total bilirubin | Direct bilirubin | ALT | AST | ALP | CEA | CA19-9 |
| --- | --- | --- | --- | --- | --- | --- | --- | --- | --- | --- |
| Collinearity statistics | Tolerance | 0.898 | 0.860 | 0.442 | 0.524 | 0.577 | 0.540 | 0.834 | 0.928 | 0.748 |
|  | VIF | 1.114 | 1.163 | 2.264 | 1.910 | 1.733 | 1.853 | 1.199 | 1.078 | 1.337 |
